# The Diversity of Cortical Inhibitory Synapses

**DOI:** 10.3389/fncir.2016.00027

**Published:** 2016-04-25

**Authors:** Yoshiyuki Kubota, Fuyuki Karube, Masaki Nomura, Yasuo Kawaguchi

**Affiliations:** ^1^Division of Cerebral Circuitry, National Institute for Physiological SciencesOkazaki, Japan; ^2^Department of Physiological Sciences, The Graduate University for Advanced Studies (SOKENDAI)Okazaki, Japan; ^3^Japan Science and Technology Agency, Core Research for Evolutional Science and TechnologyTokyo, Japan; ^4^Laboratory of Neural Circuitry, Graduate School of Brain Science, Doshisha UniversityKyoto, Japan; ^5^Department of Mathematics, Kyoto UniversityKyoto, Japan

**Keywords:** inhibitory synapse, spine, pyramidal cell, dually innervated spine, veto, thalamocortical fiber, cortex

## Abstract

The most typical and well known inhibitory action in the cortical microcircuit is a strong inhibition on the target neuron by axo-somatic synapses. However, it has become clear that synaptic inhibition in the cortex is much more diverse and complicated. Firstly, at least ten or more inhibitory non-pyramidal cell subtypes engage in diverse inhibitory functions to produce the elaborate activity characteristic of the different cortical states. Each distinct non-pyramidal cell subtype has its own independent inhibitory function. Secondly, the inhibitory synapses innervate different neuronal domains, such as axons, spines, dendrites and soma, and their inhibitory postsynaptic potential (IPSP) size is not uniform. Thus, cortical inhibition is highly complex, with a wide variety of anatomical and physiological modes. Moreover, the functional significance of the various inhibitory synapse innervation styles and their unique structural dynamic behaviors differ from those of excitatory synapses. In this review, we summarize our current understanding of the inhibitory mechanisms of the cortical microcircuit.

The microcircuit of the neocortex is a very complex, composed of excitatory neurons (including pyramidal cells and spiny stellate cells), inhibitory non-pyramidal interneurons (Jones, 1975; Kawaguchi and Kubota, 1997; Somogyi et al., 1998; Markram et al., 2004; DeFelipe et al., 2013), glutamatergic afferent axons arising from other cortical areas and from subcortical structures such as the thalamus (Jones, 2001; Kuramoto et al., 2009, 2015) as well as non-glutamatergic neuromodulatory afferents from many different brainstem nuclei (Gulledge and Stuart, 2005; Puig et al., 2014, 2015).

The GABAergic non-pyramidal cells, which function as cortical inhibitory interneurons, are located in all cortical layers, and send their axons to nearby areas or across layers or areas somewhat larger than their dendritic fields. At least ten or more GABAergic non-pyramidal cell subtypes had been identified, each with a unique form of axonal arborization (Kawaguchi and Kubota, 1997; Howard et al., 2005; Gonchar et al., 2007; Uematsu et al., 2008; Xu et al., 2010; Kubota et al., 2011; Kubota, 2014; Jiang et al., 2015; Markram et al., 2015) which innervate different domains: axon initial segment, soma, dendritic shaft and spine, of different subtypes of the pyramidal and non-pyramidal cells (Figures 1, 2; Somogyi, 1989; Kawaguchi and Kubota, 1998; Kubota et al., 2007, 2015; Jiang et al., 2013). So-called “basket” cells prefer somata as their synaptic target (Figure 3). They include parvalbumin (PV) positive fast spiking (FS) basket cells (Figures 3A,D–H), non-FS cholecystokinin (CCK) positive large basket cells (Figure 3C) which have a large perikaryon, and non-FS vasoactive intestinal polypeptide (VIP)/corticotrophin releasing factor (CRF)/CCK positive small basket cells with a small perikaryon and small dendritic and axonal fields which innervate inhibitory non-pyramidal and/or pyramidal cells (Peters, 1990; Kawaguchi and Kubota, 1996; Dalezios et al., 2002; Karube et al., 2004; Staiger et al., 2004; Uematsu et al., 2008; Kubota et al., 2011; Hioki et al., 2013; Pfeffer et al., 2013; Pi et al., 2013). In addition to the basket cells, alpha actinin 2 (AA2) positive late spiking (LS; Figure 3B) neurogliaform cells also prefer somata as their synaptic target (Price et al., 2005; Kubota et al., 2007, 2011; Uematsu et al., 2008). The other subtypes include PV positive FS chandelier cells which innervate almost exclusively axon initial segments of pyramidal cells (Kawaguchi and Kubota, 1998), non-FS somatostatin (SOM)/neuropeptide Y (NPY) positive Martinotti cells with ascending axons innervating apical (Silberberg and Markram, 2007) or apical tuft dendritic structures of the pyramidal cells (Wang et al., 2004; Jiang et al., 2013), non-FS VIP/calretinin (CR)/CRF/CCK positive double bouquet cells with descending axons innervating pyramidal cell and/or GABAergic non-pyramidal cells (Figure 1; Kawaguchi and Kubota, 1996, 1997; Karube et al., 2004; Uematsu et al., 2008; Kubota et al., 2011; Kubota, 2014). Other than the PV and somatostatin subgroups, all other subgroups appear to express 5-HT_3A_ receptor (Lee et al., 2010; Rudy et al., 2011).

**Figure 1:**
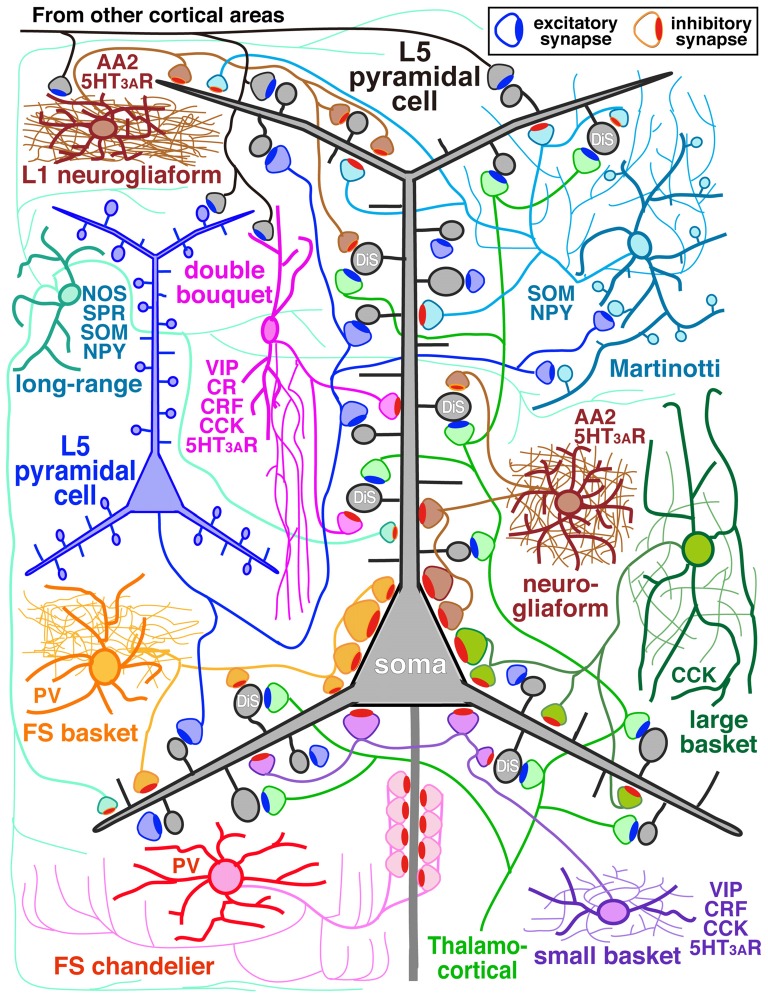
**Diagram of cortical microcircuit showing the major subtypes of GABAergic inhibitory interneurons and their synaptic diversity.** DiS, dually innervated spine; L5, layer V; AA2, alpha actinin 2; CCK, cholecystokinin; CR, calretinin; CRF, corticotrophin releasing factor; NOS, nitric oxide synthase; NPY, neuropeptide Y; PV, parvalbumin; SOM, somatostatin; SPR, substance P receptor; VIP, vasoactive intestinal polypeptide; 5HT_3A_R, 5-HT_3A_ receptor.

**Figure 2:**
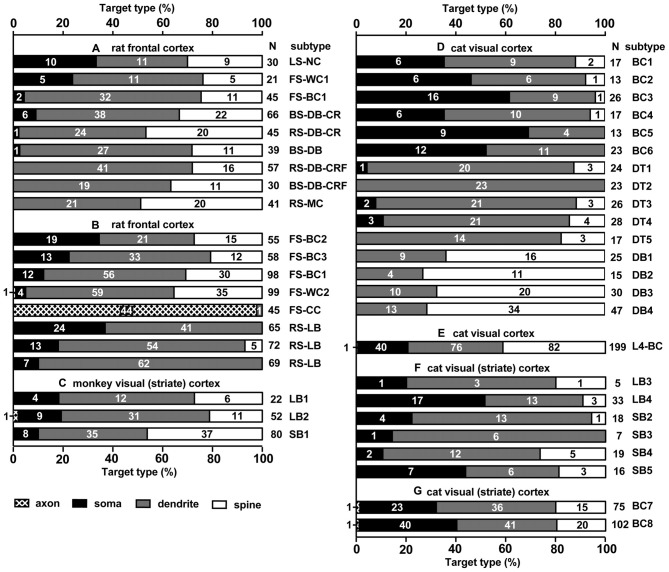
**Summary of target structures innervated by different subtypes of cortical inhibitory non-pyramidal cells; in different cortical areas and different species.** Each bar corresponds to a single analyzed neuron. Fast spiking (FS)-BC1 cell in **(A,B)** is the same cell, but analyzed at different occasions and methods. The numbers in the bar chart indicate the number of synaptic boutons analyzed on each type of postsynaptic structure. Total number of analyzed boutons are shown at the right of each bar chart (N). Most spines receiving an inhibitory synapse are dually innervated spines. LS, late spiking; FS, fast spiking; BS, burst spiking; RS, regular spiking; NC, neurogliaform cell; WC, wide arbor cell; BC, basket cell; DB, double bouquet cell; MC, Martinotti cell; CC, chandelier cell; LB, large basket cell; SB, small basket cell (including Clutch cell); DT, dendrite targeting cell; CR, calretinin; CRF, corticotropin releasing factor. **(A)** Rat frontal cortex is adapted from Kubota et al. (2007), **(B)** Rat frontal cortex (Kawaguchi and Kubota, 1998), **(C)** Monkey visual cortex (V1; Kisvárday et al., 1986), **(D)** Cat V1 (Tamás et al., 1997a), **(E)** Cat V1 (Kisvárday et al., 1987), **(F)** Cat V1 (Somogyi and Soltész, 1986), **(G)** Cat V1 (Somogyi et al., 1983).

**Figure 3:**
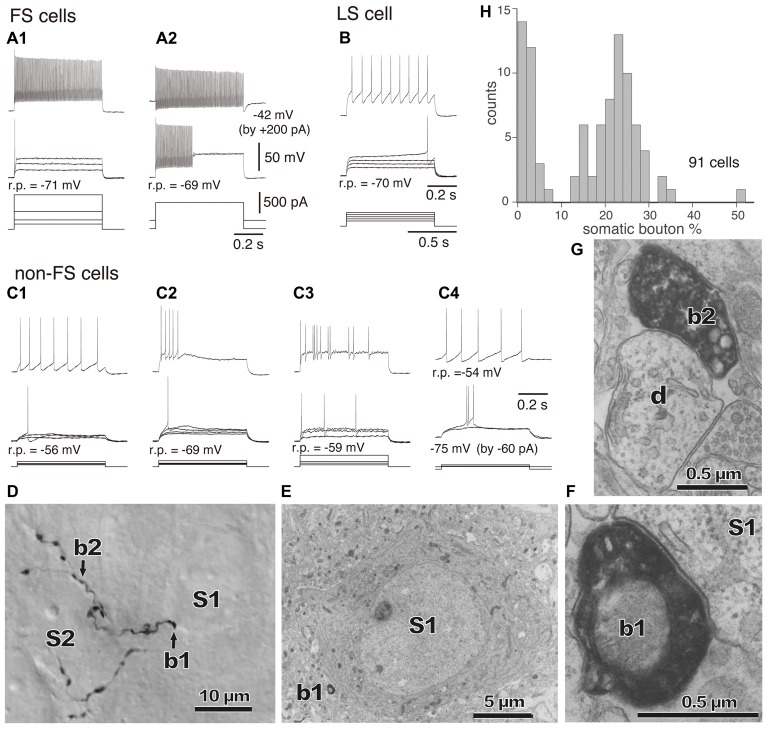
**(A–C)** Firing responses of nonpyramidal cells induced by depolarizing currents. r.p., Resting potential. DC injected into neuron to change membrane potential is indicated in parentheses. **(A1,A2)** FS cells. **(B)** A LS cell. **(C1–C4)** Non-FS cells. **(D)** Boutons of an FS basket cell (b1, b2) and their appositions on unstained somata (S1, S2) observed with differential interference contrast. **(E)** Electron micrograph of synaptic target unstained soma (S1) of the identified bouton (**D**, b1). **(F)** Synaptic contact of the bouton (b1) apposition on the soma (S1). **(G)** Electron micrographs of the bouton b2 and target dendrite (d). **(H)** Distribution of the somatic bouton percentage of nonpyramidal cells. Nonpyramidal cells were divided into cells with a low and high proportion of somatic boutons. Adapted from Karube et al. (2004).

In addition, some GABAergic cortical cells project to other areas of cortex in a “long-range” manner serving to coordinate large-scale network activity of the microcircuit with distant cortical areas (Figure 1; Tomioka et al., 2005; Caputi et al., 2013; Endo et al., 2016). They express substance P receptor (neurokinin 1 receptor)/neuronal nitric oxide synthase (nNOS)/SOM/NPY (Kubota et al., 1994, 2011; Endo et al., 2016) and have extensive axonal arborizations around the dendritic field with axonal branches extending a long distance vertically into all the layers and horizontally into other cortical areas. For instance the cells in the primary visual cortex (V1) extend their axon fibers into the secondary V1, retrosplenial cortex and across the subcortical white matter to the subiculum (Kubota et al., 1994, 2011; Endo et al., 2016). They lose dendritic spines during development (Kubota et al., 2011).

The different inhibitory non-pyramidal cell subtypes have distinct activity patterns during different cortical states and unique functional roles in the active cortical microcircuit (Klausberger and Somogyi, 2008; Isomura et al., 2009; Gentet et al., 2012; Lee et al., 2013; Pala and Petersen, 2015), although they comprise just 20% of the entire cortical neuron population. For more details, see Kawaguchi and Kubota (1997) and Kubota (2014).

The pyramidal cells are glutamatergic excitatory projection cells and are located in all cortical layers except layer I (Jones, 1984). Several pyramidal cell subtypes are found in each layer (van Aerde and Feldmeyer, 2015). They have different dendritic morphologies and electrophysiological properties, project to different target areas and receive inputs from different neurons (Thomson and Bannister, 2003). For instance, there are at least three pyramidal cell subtypes in the rodent layer 5 (L5), the crossed-corticostriatal (CCS) cells with slender-tufted apical dendrites, the corticopontine (CPn) cells with thick-tufted dendrites and the corticocortical non-striatal-projecting (CCnS) cells with slender-tufted dendrites (Wilson, 1987; Markram et al., 1997; Morishima and Kawaguchi, 2006; Brown and Hestrin, 2009; Morishima et al., 2011; Shepherd, 2013; Kim et al., 2015; Ramaswamy and Markram, 2015; van Aerde and Feldmeyer, 2015). The L5 CCS cells send their axons to ipsi-/contralateral striatum and to other ipsi- or contralateral cortical areas (Wilson, 1987), and receive inputs from layer II/III pyramidal cells, L5 CCS cells, L5 CPn cells and thalamus (Morishima et al., 2011; Hirai et al., 2012; Kim et al., 2015). On the other hand, the L5 CPn cell projects to ipsilateral striatum, thalamus, pons and spinal cord, and receives input from pyramidal cells in layer II/III, L5 CCS cells, CPn cells, basal forebrain and thalamus (Kaneko et al., 2000; Hirai et al., 2012; Ueta et al., 2013, 2014; Kim et al., 2015). The L5 CCnS cells show oval soma shape and significantly higher input resistance (263 or 249 MΩ) than the other subpopulations (CCS: 121 or 144 MΩ, CPn: 146 or 90 MΩ; Kim et al., 2015; van Aerde and Feldmeyer, 2015). This subtype in primary V1 innervates local cortex, but not striatum, and receives inputs from V1, thalamus and basal forebrain (Kim et al., 2015). Different pyramidal subtypes are likely to have different functional roles in the cortical microcircuit (Morita et al., 2012; Li et al., 2015; Lur et al., 2016).

The cortical microcircuit must be regulated and orchestrated exquisitely in each different cortical state, with a well coordinated ensemble of neuronal activities, among the diverse neuronal elements described above. Each of the inhibitory non-pyramidal cell subtypes has its own role in regulating the activity of the cortical microcircuit. For example, the coordination of network activity by gamma oscillations by PV or SOM cells (Cardin et al., 2009; Sohal et al., 2009; Kuki et al., 2015), the fine tuning of pyramidal cell activity with sensory inputs by the Martinotti/SOM cells (Murayama et al., 2009; Gentet et al., 2012), the disinhibition of pyramidal cell activity via excitatory inputs from the other cortical area by 5-HT_3A_ receptor positive cells (Lee et al., 2013). The various inhibitory neurons also make very important contributions to cortical development, memory and synaptic plasticity associated with the critical period in the visual system (Hensch, 2005; Donato et al., 2013, 2015), as well as the acquisition and retention of new memories (Hensch, 2005; Donato et al., 2013, 2015). A deficit or abnormality in the cortical inhibitory systems may be one of the main causes of neuropsychiatric disorders such as epilepsy, autism, schizophrenia, or depression. Cortical non-pyramidal cell subtypes may be the key to the pathology associated with neurological disorders (Rubenstein and Merzenich, 2003; Lewis, 2011; Curley and Lewis, 2012; Lewis et al., 2012; Hunt et al., 2013; Sauer et al., 2015).

The domain specific selectivity of cortical inhibition is expressed as four different innervation styles: axo-somatic, axo-dendritic, axo-spinous or axo-axonic innervations, and the targets could include soma, axon, dendritic spines, proximal large dendrites and distal small dendrites on excitatory or inhibitory neurons (Figures 1, 2, 3, 4, 5; Somogyi, 1989; Somogyi et al., 1998; Thomson and Bannister, 2003; Kubota et al., 2007, 2015; Kubota, 2014). Each innervation style plays a specific role in the cortical microcircuit. This review surveys recent advances in our knowledge of cortical inhibitory mechanisms, especially inhibitory synapses on different domains of the target neuron: soma, dendritic shaft, dendritic spine and axon initial segments.

**Figure 4:**
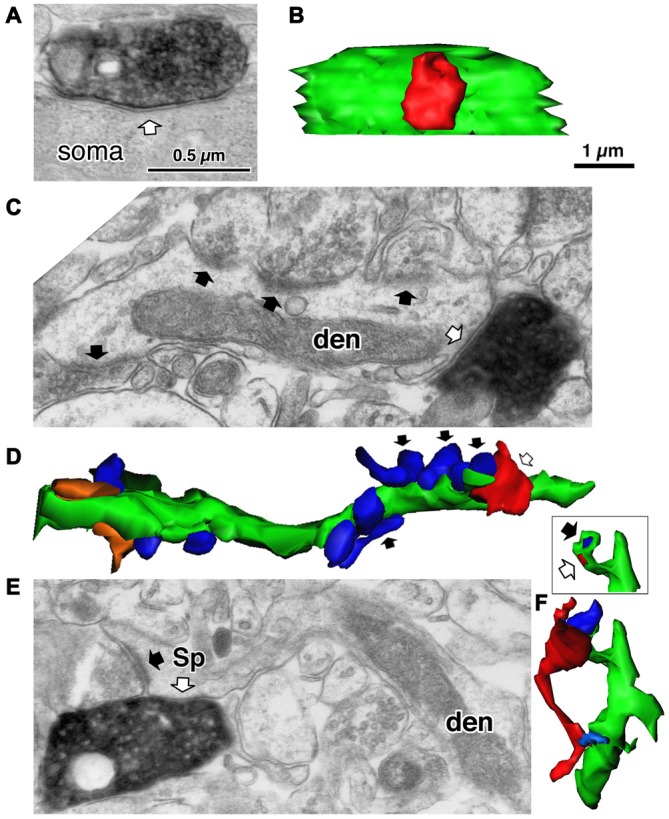
**Three different target structures of the inhibitory synapses. (A)** A symmetrical synapse (white arrow) of the LS neurogliaform (LS-NG) cell onto a postsynaptic soma. **(B)** 3D reconstructed image of the synaptic structure shown in **(A)**. The axonal terminal of the LS NG cell (red) innervates the soma (green). **(C)** A symmetrical synapse (white arrow) from a corticotropin releasing factor (CRF) positive double bouquet cell onto a dendritic shaft (den) with frequent asymmetrical inputs (black arrows). **(D)** 3D reconstructed image (green) of the dendrite shown in **(C)**. Arrows indicate the same axon terminal boutons shown in **(C)**. Red is an axon terminal of the double bouquet cell. Blue structures are boutons forming asymmetrical synapses. Orange structures are boutons forming symmetrical synapses. **(E)** A symmetrical synapse (white arrow) from a CR positive double bouquet cell onto a spine head (Sp), which is also innervated by an asymmetrical synapse (black arrow). Scale in **(A,C,E)** shares the scale bar shown in **(A)**. **(F)** 3D reconstructed image of synaptic structure shown in **(E)**. An axon terminal of the double bouquet cell (red) innervates the spine head (green), which is also innervated by an asymmetrical synaptic terminal (blue). Inset shows the two synaptic junctions (red and dark blue) on the spine, without the presynaptic boutons. Scale in **(B,D,F)** share the scale bar shown in **(B)**. Adapted from Kubota et al. (2007).

**Figure 5:**
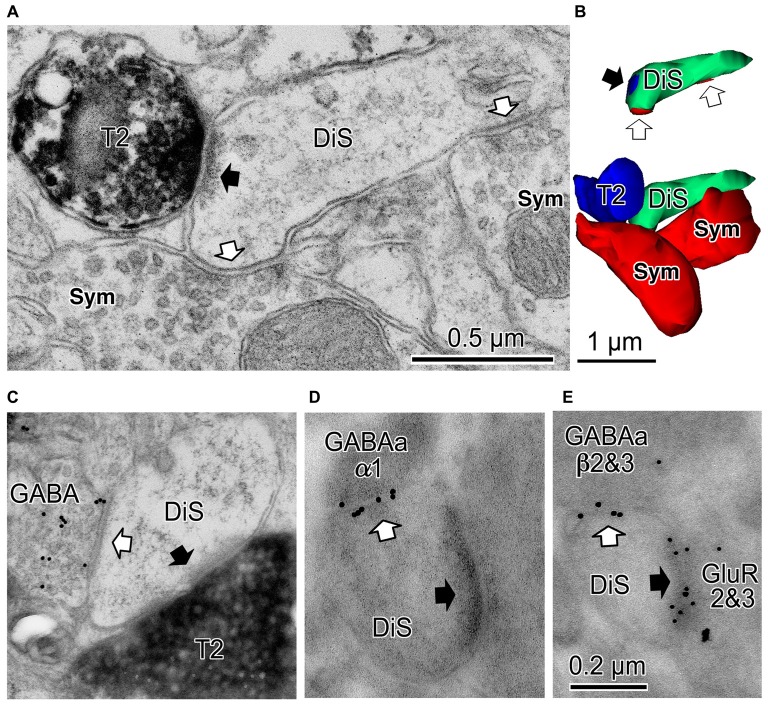
**Cortical dually innervated spines. (A)** A cortical spine (DiS) was co-innervated by a vesicular glutamate transporter 2 (VGLUT2)-positive (T2) asymmetrical synaptic terminal (black arrow) and two symmetrical synaptic terminals (Sym) (white arrows). **(B)** 3D reconstructed image of the spine and presynaptic terminals shown in **(A)**. Bottom image is the VGLUT2-positive bouton (T2, blue) contacting the spine head (DiS, green) with a synaptic junction. This spine is also innervated by two symmetrical inputs (Sym, red). Upper image shows synaptic junctions (black arrow for VGLUT2 synapse (blue) and white arrows for symmetrical synapses (red)) on the spine head (DiS, green). **(C)** A spine head (DiS) innervated by both a VGLUT2-positive (T2) asymmetrical synaptic terminal (black arrow) and a GABA-positive (colloidal gold particle labeled) symmetrical synaptic terminal (white arrow). **(D)** A spine head (DiS) innervated by an asymmetrical synaptic terminal (black arrow) and also innervated by a symmetrical synaptic terminal (white arrow). Gold particles label GABA_A_ α1 receptor subunits localized along the synaptic junction of the symmetrical synapse. **(E)** A spine head (DiS) innervated by an asymmetrical synaptic terminal (black arrow) and also innervated by a symmetrical synaptic terminal (white arrow). Larger gold particles (15 nm) labeled GABA_A_ β2&3 receptor subunits localized along the synaptic junction of the symmetrical synaptic terminal and smaller particle (10 nm) labeled glutamate receptor GluR2&3 subunits of the associated asymmetrical synaptic junction. Scale in **(C)** shares the scale bar shown in **(A)**, and the scale in **(D)** is the same as shown in **(E)**. Adapted from Kubota et al. (2007).

## Axo-Somatic Inhibition

The strong axo-somatic inhibition is the basis for the “classical” concept of inhibition in the cortical microcircuit (Somogyi, 1989; Kawaguchi and Kubota, 1996, 1998; Somogyi et al., 1998; Kubota et al., 2007, 2015; Kubota, 2014), however quantitatively axo-somatic contacts are just a small fraction of the total synaptic output of inhibitory non-pyramidal cells (for instances: 7% and 12% of the inhibitory synaptic contacts in the cat and monkey primary visual cortices, respectively; Figure 2; Beaulieu and Colonnier, 1985; Beaulieu et al., 1992). Even among the axon terminals of a basket cell, the axo-somatic terminals comprise only 10–37% in rat frontal cortex (Kawaguchi and Kubota, 1998), 10–70% in cat striate cortex (Somogyi et al., 1983; Kisvárday et al., 1985, 1987; Somogyi and Soltész, 1986; Tamás et al., 1997a), and 10–18% in monkey striate cortex (Kisvárday et al., 1986; Figure 2). Clearly the axo-somatic terminal represents only a fraction of the basket cell terminals. About half of the non-pyramidal cell subtypes innervate somata of target cells to various degrees. The FS basket cell, the small basket cell, the large basket cell, and the neurogliaform cell innervate somata frequently (Figures 1, 2; Karube et al., 2004; Kubota et al., 2007; Uematsu et al., 2008; Kubota, 2014). The FS basket cell, which expresses PV, and the large basket cell, which expresses CCK and the cannabinoid receptor (CB1R), respond differently to repetitive signals with a mechanism exquisitely regulated by cannabinoids (Hefft and Jonas, 2005; Glickfeld and Scanziani, 2006; Freund and Katona, 2007). The FS basket cells respond reliably and immediately to repetitive signals, whereas the CCK expressing large basket cells respond to repetitive signals with depression and delay in the hippocampus (Glickfeld and Scanziani, 2006). Interestingly CCK enhances the FS basket cell activity and induces endocannabinoid-mediated inhibition of GABA release from CCK/CB1R expressing basket cell terminals in the hippocampus (Foldy et al., 2007). During theta oscillations *in vivo*, the FS basket cells fire on the descending phase, whereas the CCK expressing large basket cells fire on the ascending phase (Klausberger et al., 2003, 2005). This is a typical example of how different non-pyramidal cell subtypes have different functional roles in the cortical microcircuit.

**Figure 6:**
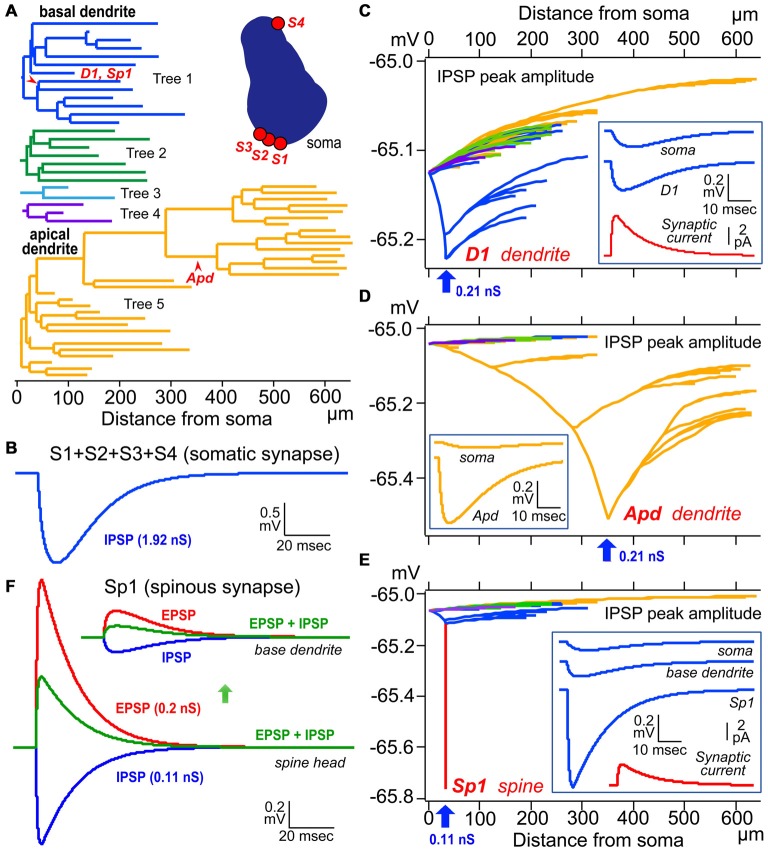
**Simulation analysis of inhibitory postsynaptic potential (IPSP) on dendritic spine, shaft and somatic IPSCs. (A)** Left, color-coded dendrogram with inhibitory synaptic current injection sites on the dendrites (red arrowheads). Right, a cross-section of the cell body of the model pyramidal cells with four somatic synaptic contacts. **(B)** The simulated IPSP wave-form at the soma induced by injection of a total of 1.92 nS inhibitory conductance summed across the somatic synapses S1–S4. **(C)** Peak dendro-somatic potential changes (color-coded as in **A**) induced by an inhibitory conductance of 0.21 nS (inset, red trace, simulated synaptic current) injected into the shaft of a basal dendritic branch (D1, blue arrow; corresponding to D1 in **A**). The simulated IPSP wave-forms at the injection site (D1) and soma are shown in the inset (blue traces). Resting potential is −65 mV. **(D)** Peak dendro-somatic potential changes induced by an inhibitory conductance of 0.21 nS (simulated IPSC is quite similar to the red trace shown in inset in **C**, red trace) injected into the shaft of an apical dendritic branch (Apd, blue arrow; corresponding to Apd in **A**). The simulated IPSP wave-forms at the injection site (Apd) and soma are shown in the inset (yellow traces). Resting potential is −65 mV. **(E)** Peak membrane potential changes over the somato-dendritic membrane induced by an inhibitory conductance of 0.11 nS injected at the spine head Sp1 (blue arrow, corresponding to Sp1 in **A**; simulated IPSC shown in red trace in inset). Peak inhibitory potential in the spine is indicated in red. The simulated IPSP wave-forms at the injection site (Sp1), base of dendrite and soma are shown in inset (blue traces).** (F)** Reduction (green) of the excitatory postsynaptic potential (EPSP) resulting from the injection of an EPSC waveform (with peak conductance of 0.2 nS; red) at the Sp1 spine head (lower superimposed traces) and base dendrite locus (upper superimposed traces)) by an IPSC (0.11 nS; blue) injected at the same site and time. Adapted from Kubota et al. (2015). The simulations were made with “NEURON” (Hines and Carnevale, 1997) and the CS56 pyramidal cell model neuron publicly available at ModelDB (Accession no: 183424; http://senselab.med.yale.edu/ModelDB/showModel.cshtml?model=183424. The datasets are also available at http://www.nips.ac.jp/circuit/).

The “basket cell” was defined as a cell type making many axo-somatic boutons which form pericellular nests around the pyramidal cell somata, resembling a basket (Figures 3D–F; Jones and Hendry, 1984). The axo-somatic boutons are therefore called “basket terminals”. In rat frontal cortex, the basket cells are defined as having basket terminals for >12% and typically 25% or more among all its axonal boutons (Figure 3H; Karube et al., 2004). The basket terminals innervate pyramidal cell somata (Figures 1, 2, 3) and also somata of other basket cells (Jiang et al., 2015). Autaptic self-inhibition of a basket cell on its own soma, which usually induces a large inhibitory postsynaptic potential (IPSP), is also frequently found (Tamás et al., 1997b; Deleuze et al., 2014). The inhibitory somatic synapse junction area is larger than the other inhibitory synapses and its conductance is probably <2 nS (typically 0.4–1 nS; Kubota et al., 2015). We estimated the conductance using a unit value of electric charge/synaptic junction area (343.3 fC/μm^2^) measured by a paired recording study of the FS basket cell and the L5 CCS pyramidal cell combined with 3D reconstruction of the synaptic junction from serial electron micrographs and simulation analysis (Kubota et al., 2015). The multiple somatic synapses of a single presynaptic FS basket cell clustered on a single postsynaptic pyramidal cell soma are probably activated simultaneously, because of their large probability of release, as found in hippocampal glutamatergic synapses (Holderith et al., 2012). A typical FS cell makes, on average, 3.2 ± 2.0 (range 1–13 basket terminals per postsynaptic cell soma, analysis of 112 axon terminals of 7 presynaptic FS basket cells) contacts on the soma of a postsynaptic pyramidal cell (Figures 3D–H; Karube et al., 2004). One presynaptic FS basket cell may hyperpolarize the postsynaptic pyramidal cell soma by >1.33 mV with simultaneous activation of all its axo-somatic contacts, as shown by simulation analysis with holding membrane potential −65 mV (S1–S4 in Figures 6A,B; Kubota et al., 2015). Similar unitary IPSP amplitudes (0.4–3 mV) were observed in dual recording experiments in cortical or hippocampal slices (Buhl et al., 1994; Stuart, 1999; González-Burgos et al., 2005; Thomson and Lamy, 2007; Ma et al., 2012; Mercer et al., 2012) Thus a unitary IPSP would be sufficient to cancel an unitary excitatory postsynaptic potential (EPSP) induced by a single spike from a presynaptic pyramidal cell, because the EPSP amplitude is in a similar range (0.3–5.9 mV, Table 1 in Thomson et al., 2002; Feldmeyer et al., 2005; Frick et al., 2007, 2008; Thomson and Lamy, 2007). Multiple FS basket cells nearby may fire simultaneously due to frequent connection via gap junctions and mutual innervations (Amitai et al., 2002; Fukuda et al., 2006; Hu et al., 2011; Otsuka and Kawaguchi, 2013), so the postsynaptic pyramidal cell could be inhibited via much deeper hyperpolarization and/or via shunting inhibition mediated by synaptic high GABA_A_ conductance under physiological conditions. This would counteract strong excitation and may prevent the pyramidal cell from firing (Kubota et al., 2015). In conclusion, the somatic GABAergic synapse has very strong inhibitory effect on target cell activity, although axo-somatic boutons comprise only a small fraction (a few to 10%) of all GABAergic synapses found on one postsynaptic neuron (Ahmed et al., 1997; Mátyás et al., 2004).

## Axo-Dendritic Inhibition

Except the chandelier/axo-axonic cells, all the inhibitory non-pyramidal cells in the rat frontal cortex innervate the dendritic shaft as their major target domain. Axo-dendritic boutons comprise 37–90% of the total synaptic contacts of the individual inhibitory non-pyramidal cell (Kawaguchi and Kubota, 1998; Kubota et al., 2007; Figures 1, 2, 3G, 4C,D). The inhibitory dendritic synapse junction area is smaller than the somatic inhibitory synapses and its estimated conductance is <1 nS (typically 0.1–0.8 nS; Kubota et al., 2015). The synaptic junction area size is well correlated with the target dendrite size, which probably provides an effective impedance matching (Kubota and Kawaguchi, 2000; Kubota et al., 2015). In our simulation analysis, inhibitory synaptic conductance (0.21 nS) injection into the basal dendritic shaft of a postsynaptic pyramidal cell held at a membrane potential of −65.0 mV (D1 in Figures 6A,C, 34 μm from soma) hyperpolarized the local dendrite by 0.23 mV. The hyperpolarization attenuated steeply in the proximal direction but relatively gently in the distal direction (Figure 6C), similar to the spread of an EPSP (Gidon and Segev, 2012). It caused a hyperpolarization of 0.13 mV at the soma (Figure 6C). The same inhibitory synaptic conductance (0.21 nS) injection on a further distal dendritic shaft located in the apical dendritic branch (Apd in Figure 6A, 350 μm from soma) hyperpolarized the local dendrite by 0.51 mV, but attenuated greatly at a distance (Figure 6D), hyperpolarizing the somatic membrane potential by only 0.04 mV (Figure 6D). These simulation analyses indicate that the inhibitory synapse on dendrites effectively hyperpolarizes the membrane potential only in the local dendritic domain. In addition, assuming more excitatory synaptic conductance *in vivo*, the inhibitory synaptic current would not spread any further. This assumes a only a simple inhibitory mechanism, although GABA shunting inhibition, voltage dependent conductance and so on might also be considered from a physiological point of view (Song et al., 2011; Doyon et al., 2016). In apical dendrites of pyramidal cells in layer 2/3 of the binocular region in the mouse primary V1, spine density is 4.42/10 μm, density of inhibitory synapses along the apical dendritic shaft is 1.68/10 μm and density of inhibitory synapses on spines is 0.71/10 μm. Densities are quite similar on basal dendrites (Chen et al., 2012; Villa et al., 2016). Usually pyramidal cell spine receives only one excitatory synapse, and almost all excitatory synapses on pyramidal cells are on spines (Chen et al., 2012), therefore the ratio of excitatory to inhibitory synapses on the dendrites can be about 2:1.1. *In vivo*, excitatory and inhibitory inputs are dynamically balanced in individual neurons from moment to moment (Xue et al., 2014). Stronger inhibition indicates simultaneously larger excitatory inputs. The excitatory conductance increase at dendrites would limit the spatial spread of increased inhibition thereby spatially confining the dendritic inhibitions (Doyon et al., 2016).

## Axo-Spinous Inhibition of Dually Innervated Spines

Most inhibitory cells also innervate dendritic spines of pyramidal cells. The chandelier and CCK positive large basket cells are the only subtypes which do not target the spine (Figures 1, 2; Kawaguchi and Kubota, 1998). Inhibitory synapses on spines were discovered by electron microscopic observation of the cat cortex, and each recipient spine was found to receive also an asymmetrical (excitatory) synapse (Jones and Powell, 1969), so these spines were called dually innervated spines (DiS). Axo-spinous synapses originate from diverse non-pyramidal cell subtypes in different species: rodent, cat, monkey and human cerebral cortex. About 20–70% of the axon terminals of the cortical non-pyramidal cells target spines (Figures 1, 2, 4E,F, 5; Somogyi and Cowey, 1981; Somogyi et al., 1983; Kisvárday et al., 1985, 1987, 1990; Somogyi and Soltész, 1986; Tamás et al., 1997a; Kubota et al., 2007). The DiSs were found in the diverse cortical areas; frontal (Kubota et al., 2007, 2015), visual (Chen et al., 2012; van Versendaal et al., 2012; Villa et al., 2016) and somatosensory cortex (Knott et al., 2002; Isshiki et al., 2014), as well as in subcortical areas (Wilson et al., 1983; Ingham et al., 1998). This suggests that the inhibition targeting spines is a widespread motif in the microcircuitry of the brain.

The excitatory asymmetrical synaptic input on the cortical DiS originates from thalamocortical excitatory axon terminal that expresses vesicular glutamate transporter 2 (VGLUT2; (Figures 5A–C; Kubota et al., 2007). The excitatory afferents in the rodent frontal cortex are probably from the ventral anterior-ventral lateral (VA/VL) or ventral medial (VM) motor thalamic nuclei (Kuramoto et al., 2009, 2015; Shigematsu et al., 2015). The DiSs are large in volume and frequently receive a perforated synapse (Figure 5C). The inhibitory synapse of the DiS expresses GABA_A_ alpha 1 and beta 2&3 receptors and the excitatory synapse has AMPA GluR2&3 receptors (Figures 5D,E; Kubota et al., 2007). These findings suggest that the DiS is a mature, stable spine (Matsuzaki et al., 2001; Holtmaat et al., 2006; Isshiki et al., 2014; Villa et al., 2016). The inhibitory synapse on the spine should efficiently veto the thalamic excitatory input and may reduce the probability of pyramidal cell firing (Sun et al., 2006; Gulledge et al., 2012; Chiu et al., 2013; Kubota et al., 2015). The largest proportion of spines with inhibitory synapses was found in layer 1 (16.3% of all VGLUT2 positive (thalamic) recipient spines). Towards the deeper layers, the fraction of VGLUT2-recipient spines which are dually innervated is gradually reduced (layer 2/3: 14.5%, layer 4: 8.7%, layer 5: 6.8% and layer 6: 4.4%; Kubota et al., 2007). The estimated fraction of DiS among all spines in each layer is <10% in layer 1, <3% in layer 2/3, <2% in layer 5 and <1% in layer 6.

The junction area of inhibitory spine synapse is similar or smaller than the dendritic inhibitory synapse, and its estimated conductance is <0.5 nS (typically 0.05–0.3 nS; Kubota et al., 2015). The synaptic junction area is well correlated with the target spine head volume as with the target dendrite (Kubota et al., 2015). Inhibitory synaptic conductance (0.11 nS) injected into the spine head (Sp1 in Figures 6A,E) resulted in a strong 0.78 mV hyperpolarization of the target spine head, but only 0.12 mV was conducted to the base dendritic region (Figure 6E). This indicates that the spine head is well isolated electrotonically by the high resistance of the spine neck (500 MΩ; Harnett et al., 2012). To analyze the effect of the IPSP on an EPSP at the DiS, 0.2 nS excitatory synaptic conductance in addition to the 0.11 nS inhibitory synaptic conductance were injected in the spine head. The excitatory current depolarized the local spine head by 1.3 mV and the inhibitory current reduced the EPSP by about a half (Figure 6F). Therefore the inhibitory spine synapse can effectively veto the local excitatory synaptic input, at least blocking NMDA receptor activation (Figure 6F; Gulledge et al., 2012; Chiu et al., 2013).

## Strong Axo-Dendritic/Axo-Spinous Net Inhibition

Although the individual inhibitory axo-dendritic/axo-spinous synapses produce only a small local inhibitory conductance in comparison with the axo-somatic synapses, the local single-bouton IPSP in the dendrite is not any smaller than the local IPSP in the soma, and their integrated inhibition is powerful (Cossart et al., 2001; Gentet et al., 2012). Axo-dendritic inhibition is the major inhibition style among the cortical GABA synapses. About 90% or more of the cortical inhibitory synapses are axo-dendritic/axo-spinous (Ahmed et al., 1997; Mátyás et al., 2004). About 5–20 axonal boutons of one non-pyramidal cell usually contact different, distant dendrites of the target pyramidal cell, and the resulting hyperpolarizations may sum poorly (Kubota et al., 2015). Even so, the scattered inhibitory synapses on different dendritic branches can effectively reduce the integrated EPSP amplitude conducted to the soma if inhibitory cells fire at frequencies of 40–50 Hz (Isomura et al., 2009). Together with the GABA shunting effects (Gidon and Segev, 2012), axo-dendritic/axo-spinous inhibition can inhibit the spiking activity of the postsynaptic target pyramidal cell (Cossart et al., 2001; Gentet et al., 2012; Kubota et al., 2015). The inhibition of individual dendritic branches and/or spines may also have the advantage of inhibiting a functionally specific excitatory signal on the dendritic domain (Chen et al., 2013), which is in marked contrast to somatic inhibition, which inhibits all incoming excitation non-specifically.

## Different Forms of GABA Mediated Inhibition

The cortical inhibition styles mentioned above mostly involve ionotropic GABA_A_ receptors, which have a rapid synaptic event lasting only a few milliseconds and are mediated by Cl^−^ ion flow through the synaptic channels. On the other hand, extrasynaptically located metabotropic GABA_B_ receptors mediate very slow IPSCs lasting for tens of milliseconds or occasionally even longer (Kawaguchi, 1992; Tamás et al., 2003; Schwenk et al., 2010) and these slow IPSCs arise from non-synaptic volume transmission (Oláh et al., 2009) or high GABA release by burst firing of a presynaptic interneurons (Thomson and Destexhe, 1999). They also mediate inhibition by postsynaptic NMDA receptor mediated Ca^2+^ influx reduction acting via the downregulation of cyclic adenosine monophosphate (cAMP) and protein kinase A (PKA), and by suppressing transmitter glutamate release by inhibition of presynaptic voltage-sensitive Ca^2+^ channels and/or by activation of K^+^ channels (Chalifoux and Carter, 2011; Lur and Higley, 2015; Urban-Ciecko et al., 2015). The apical tuft dendrites of the L5 pyramidal cell in layer 1 are inhibited by two different microcircuits using GABA_A_ or GABA_B_-receptors (Palmer et al., 2012a). The first one uses exclusively GABA_A_-receptors and mediates feedback inhibition via the Martinotti cell after *in vivo* contralateral hindlimb stimulation. This circuit controls the sensitivity and dynamic range of the L5 pyramidal cell (Murayama et al., 2009). The first excited L5 pyramidal cell recruits the Martinotti cells to inhibit the tuft dendrites of the neighboring L5 cells as a surround inhibition (Palmer et al., 2012a). The second inhibitory circuit consists of the AA2 positive neurogliaform cells in layer I, excited by callosal excitatory axonal fibers activated by ipsilateral hindlimb stimulation. The AA2 positive neurogliaform cells inhibit the apical tuft dendrites of the L5 pyramidal cells with GABA_B_-mediated inhibition, and reduce their spiking activity by 25% in comparison to the control condition of the contralateral hindlimb stimulation only (Palmer et al., 2012b). These results illustrate how the different forms of inhibition in different cortical microcircuits are exquisitely used in regulating cortical activity in the living body.

Furthermore, a different form of inhibition, shunting inhibition, may suppress the excitatory signal more efficiently. Shunting inhibition attenuates the EPSP divisively, by reduction in input resistance of the postsynaptic membrane rather than by hyperpolarizing the postsynaptic membrane potential. This works effectively at neuronal domains where the membrane resting potential is similar to the inhibitory synaptic reversal potential (Gulledge and Stuart, 2003; Song et al., 2011), and the inhibitory synapse situated on the conduction pathway of EPSP to the action potential initiation site (Hao et al., 2009). The shunting is largely confined to the same branch and high for inhibitory synapses located in distal dendritic branches (Hao et al., 2009). A simulation analysis by Gidon and Segev (2012) suggested that shunting can spread beyond the anatomical domain demarcated by the inhibitory synapses, can effectively counteract the excitatory current generated in the nearby dendritic domain, even under higher excitation/inhibition ratios (>2; Megías et al., 2001; Merchán-Pérez et al., 2009; Gidon and Segev, 2012).

## *In Vivo* Structural Dynamics of the Inhibitory Synapses

The cortical inhibitory synapse has its own structural dynamics which is different from that of the cortical excitatory synapse. Strong thalamic input during whisker stimulation for 24 h increases the number of DiS in somatosensory cortex (Knott et al., 2002). Monocular deprivation, a model of sensory input-dependent plasticity, induces loss of inhibitory dendritic shaft and spine synapses in the primary V1 (Chen et al., 2012; van Versendaal et al., 2012). The inhibitory synapses on DiS are more dynamic than the inhibitory synapses on the dendritic shaft or excitatory synapses on spines. They frequently exhibit recurrent dynamics, i.e., repetitive appearance and disappearance of inhibitory synapses on the DiS, under daily *in vivo* imaging, even in normal physiological circumstances. Under the same conditions, the excitatory synapses on the host spines remained stable (Villa et al., 2016). This characteristic structural dynamics of the inhibitory synapses provides a potential mechanism for reversible gating of specific excitatory connections, such as visual input from thalamic lateral geniculate nucleus (Villa et al., 2016).

## Axo-Axonic GABA Response

Chandelier cells almost exclusively innervate the axon initial segments of pyramidal cells with vertically oriented axon-terminal bouton alignment and may target other chandelier cells as well (Figure 1; Somogyi, 1977; Jiang et al., 2015). Five to six chandelier cells may converge onto one axon initial segment of a pyramidal cell in layer 2/3 of cat striate cortex with about eight presynaptic axo-axonic terminal boutons per chandelier axonic bouton cartridge (Somogyi et al., 1982), and 3.8 ± 0.3 chandelier cells may participate in the innervation with 4.1 ± 0.2 presynaptic boutons in mouse somatosensory cortex (Inan et al., 2013). The chandelier cells do not innervate all the pyramidal cells evenly. Pyramidal cells in supragranular layers receive large number of axo-axonic synapses on the axon initial segment, 16–23 for callosal cells and 22–28 for ipsilateral corticocortical cells, whereas the corticothalamic pyramidal cells in the infragranular layers only receive 1–5 synapses in cat V1 (Fariñas and DeFelipe, 1991). The chandelier cells have been found in rodent, cat, monkey and human cortex (Somogyi, 1977; Somogyi et al., 1982; DeFelipe et al., 1989; Kawaguchi and Kubota, 1998; Szabadics et al., 2006; Inan et al., 2013; Jiang et al., 2015). Most chandelier cells express PV and are FS cells, while the others are CRF-positive (Lewis and Lund, 1990) and/or non-FS cell (Kawaguchi, 1995; Taniguchi et al., 2013). They probably suppress the target cell spiking activity by releasing the inhibitory transmitter GABA. This was shown by a local unitary field analysis of spike discharge of axo-axonic cells in the hippocampus, and by the population discharge activity timing of pyramidal and chandelier cells during theta rhythm oscillation (Klausberger and Somogyi, 2008; Glickfeld et al., 2009; Viney et al., 2013). However they may also excite the postsynaptic cell due to high Cl^−^ concentration of the intracellular fluid of the target axon which is probably achieved by a low potassium chloride co-transporter 2 (KCC2) distribution in the axon initial segment of the target pyramidal cells (Szabadics et al., 2006; Woodruff et al., 2009; Báldi et al., 2010) and Na-K-2Cl co-transporter (NKCC1) existence on the neuronal membrane for Cl^−^ uptake into intracellular space (Khirug et al., 2008). The equilibrium potential of GABA (E_GABA_) values obtained with local uncaging of GABA on axon initial segment, soma and dendrite of the dentate gyrus cells of hippocampus using gramicidin-perforated patch-clamp method were −59.4 ± 1.5 mV, −65.8 ± 1.2 mV, and −70.9 ± 1.5 mV, respectively, and the intracellular levels of Cl^−^ [Cl_i_] calculated on the basis of the E_GABA_ values were 11, 7.9 and 6.0 mM, respectively (Khirug et al., 2008). Under a resting membrane potential of −72.7 ± 2.6 mV measured using the gramicidin-perforated patch-clamp method, a marked depolarizing driving force of about 13 mV can be mathematically predicted at the axon initial segment of the target cells, and GABA action may be associated with the efflux of chloride ions and the depolarization of the axon initial segment. With more excitatory synaptic conductances *in vivo*, the membrane potential is probably more depolarized and a weak hyperpolarizing driving force can be obtained. As a result, GABA_A_ response could hyperpolarize the membrane potential at the axon initial segment by a small chloride ions influx. This is supported by *in vivo* studies or slice preparation (Klausberger and Somogyi, 2008; Glickfeld et al., 2009; Viney et al., 2013). Thus they could work in a diametrically opposite fashion depending on the circuit condition (Woodruff et al., 2011). This topic is still controversial and further studies should be done.

## Excitatory/Inhibitory GABA Response

GABA responses at dendritic segments of cortical pyramidal cells could be excitatory under certain circumstances, whereas somatic GABA responses were inhibitory when coincident with excitatory synaptic potentials (Gulledge and Stuart, 2003). The ambient GABA could excite or inhibit adult hippocampal interneurons depending on the activated conductances, with its depolarizing reversal potential (Song et al., 2011). These suggest that the excitatory or inhibitory actions of GABA are dependent not only on the reversal potential, but also on the activated conductance. In the inflammatory chronic pain model mouse, the dendritic and somatic KCC2 expression is reduced in layer 2/3 pyramidal cells of somatosensory cortex, making the GABA equilibrium potential more positive than the control (Eto et al., 2012). In this condition, the GABA response could depolarize the pyramidal cells at the resting membrane potential, −70 ~ −80 mV (Gulledge and Stuart, 2003), but GABA actions still seem to be inhibitory on the basis of the GABA_A_ receptor agonist/antagonist application studies *in vivo* (Eto et al., 2012). This suggests that GABAergic transmission could be inhibitory even in the case of a KCC2 reduction. To fully understand the GABAergic actions, we should know the dynamics of intracellular Cl^−^ concentration, maybe dependent on the domain (somata, axons and dendritic shafts/spines) and the intracellular membrane potential at each domain, and how hyperpolarization and shunting inhibitions are used within individual domains (Song et al., 2011; Doyon et al., 2016).

## Future Aspects

Some of the information on inhibitory styles in cortical microcircuits presented above has been obtained recently, using new techniques. However, we still have many unresolved questions concerning inhibition in the cortical microcircuit architecture and its functional implications. For instance, it would be interesting to know why one non-pyramidal cell innervates its target cell on different membrane domains, why inhibitory synapses on spines are recurrently dynamic, and how the diverse non-pyramidal cell subtypes exert coordinated inhibition to enable highly complex cortical activity patterns. Future studies are sure to make new and important findings on inhibitory structure and function in the cortical microcircuit.

## Ethics Statement

Animal experimentation: All surgical and animal care methods was performed in strict accordance with the Guidelines for the Use of Animals of IBRO and our institutional Animal Care and Use committee (National Institute for Physiological Sciences) with reference number 15A091. All surgery was performed under ketamine and xylazine, or isoflurane anesthesia, and every effort was made to minimize suffering.

## Author Contributions

YK: conception and design, acquisition of data or micrographs, analysis and interpretation of data, making figure panels, drafting or revising the article. FK: making figure panels, drafting or revising the article. MN: acquisition of simulation data. YK: conception and design, drafting or revising the article.

## Funding

This work was supported by a Grant-in-Aid for Scientific Research (A; 25250005), (B; 25290012), (15K14324) and on Innovative Areas “Adaptive circuit shift (No. 3603)” (26112006; 15H01456) and “Prediction and decision making (No. 4303)” (26120730) of The Ministry of Education, Culture, Sports, Science, and Technology, Japan, The NOVARTIS Foundation (Japan) for the Promotion of Science.

## Conflict of Interest Statement

The authors declare that the research was conducted in the absence of any commercial or financial relationships that could be construed as a potential conflict of interest.
